# The ımpact of pelvic girdle pain on quality of life and depressive symptoms during pregnancy

**DOI:** 10.1590/1806-9282.20250572

**Published:** 2026-01-09

**Authors:** Yurdagül Günaydin, Esma Kir, Serpil Toker, Medine Kir Deprem

**Affiliations:** 1 Tarsus University, Faculty of Health Sciences, Department of Nursing – Mersin, Turkey.; 2 Yozgat Bozok University, Faculty of Health Sciences, Department of Midwifery – Yozgat, Turkey.; 3 Tokat Gaziosmanpaşa University, Faculty of Health Sciences, Department of Midwifery – Tokat, Turkey.; 4 Kayseri City Training and Research Hospital, Department of Family Medicine – Kayseri, Turkey.

**Keywords:** Depressive symptoms, Life quality, Pelvic pain, Pregnancy

## Abstract

**OBJECTIVE:**

The aim of the study was to examine the impact of pelvic girdle pain on quality of life and depressive symptoms during pregnancy.

**METHODS:**

This descriptive, cross-sectional study involved 433 second- and third-trimester pregnant women who volunteered at two public hospitals in Central Anatolia, Turkey, between March 2024 and March 2025, excluding those with high-risk pregnancies. Data were collected using the Demographic Form, Pelvic Girdle Questionnaire, Short-Form Health Survey (SF-36), Beck Depression Inventory, and Visual Analog Scale.

**RESULTS:**

The mean age of women was 27.81±5.12. The findings revealed significant negative correlations between the Pelvic Girdle Questionnaire and SF-36 subscales, including physical function, physical and emotional role limitations, social functioning, general health and pain (p<0.05). Pelvic Girdle Questionnaire was positively correlated with depression, Visual Analog Scale, and gestational weeks (p<0.05). Moreover, 32.9% of the variance in Pelvic Girdle Questionnaire scores is explained by SF-36 subdimensions (physical function, role limitations, energy/vitality, mental health, and pain), number of births, and Visual Analog Scale.

**CONCLUSION:**

Pelvic girdle pain negatively affects physical and mental well-being, increases pain, and reduces quality of life in pregnancy. Its severity rises with gestational age and is linked to depressive symptoms.

## INTRODUCTION

During pregnancy, biochemical, physiological, and structural changes lead to significant musculoskeletal alterations^1^. Pregnancy hormones relax connective tissue, while weight gain increases mechanical load. Pregnancy-induced changes cause hyperlordosis, pelvic tilt, and shift the center of gravity, affecting lower back support^2^. Pregnancy-related musculoskeletal and postural changes can lead to pelvic girdle pain (PGP), a common complaint with prevalence ranging from 7 to 65%^3,4^. PGP primarily affects the sacroiliac joint, iliac crest, and gluteal fold, and can also involve the posterior thigh and suprapubic regions^5^. Weight gain and hormonal changes in late pregnancy can trigger PGP, increasing pain intensity. Increased pain makes daily activities such as standing, walking, and sitting more difficult, thereby causing physical limitations. Moreover, this pain reduces work productivity, significantly decreases quality of life, and increases susceptibility to chronic pain syndromes during pregnancy^6^. PGP negatively impacts pregnant women's health-related quality of life, particularly physically, psychologically, and socially^7^.

A decrease in quality of life during pregnancy can lead to depressive symptoms that negatively affect the expectant mother's mood and health. Depression is the most common psychiatric disorder during pregnancy, with prevalence ranging from 15 to 65%^8^. Depression can negatively affect not only the pregnant woman but also the developing fetus. Additionally, depressive symptoms can affect newborn care and mother-infant bonding and lead to long-term psychological and developmental consequences^9,10^. Identifying risk factors for depression, especially in pregnant women, is crucial for advancing research in this area. PGP negatively affects pregnant women's quality of life and increases depression scores. However, studies on this subject are limited. Further research on the PGP-depression relationship is crucial for developing treatment and care strategies. Because PGP has significant effects on the physical and psychological health of pregnant women, more comprehensive research is crucial. This study examined the effects of PGP on quality of life and depressive symptoms during pregnancy.

## METHODS

This descriptive and cross-sectional study was conducted between March 2024 and March 2025 in two public hospitals in Central Anatolia. Two large public hospitals located in a city in Central Anatolia provide comprehensive pregnancy and maternal healthcare services with multidisciplinary teams and experienced healthcare staff. Participants were recruited through voluntary participation during routine outpatient visits to the obstetrics and gynecology clinics. Eligible healthy pregnant women meeting the inclusion criteria were invited to join the study after being informed about its purpose.

Before starting data collection, ethical approval was granted by the Yozgat Bozok University Social and Human Sciences Ethics Committee (Approval No: 12/10, Date: 20.03.2024), and all participants signed an informed consent form after being informed about the purpose of the study. The study included healthy pregnant women aged ≥19 years in their second or third trimester attending routine outpatient obstetrics follow-ups who provided informed consent. Women with high-risk pregnancies (e.g., gestational hypertension, gestational diabetes) were excluded. The sample size was calculated using the G*Power 3.1.9.7 program. Based on Cohen's^11^ suggested medium effect size, a confidence interval of 80%^12^, and a 5% margin of error, the minimum required sample was calculated as 368 participants. Considering a potential data loss of 10%, data collection was completed with 433 pregnant women.

Data collection tools consisted of a Demographic Information Form, Pelvic Girdle Questionnaire (PGQ), Short-Form Health Survey (SF-36), and Beck Depression Inventory (BDI). The Demographic Information Form, developed by the researchers in line with the literature and expert opinions, comprised 21 items assessing sociodemographic and obstetric history, including education, employment, family type, pregnancy status, income level, timing of pregnancy onset, smoking, and alcohol use^6,7^. The PGQ includes 25 items—20 assessing activity limitations and 5 assessing symptoms—scored on a four-point scale. Total scores range from 0 to 100, with higher scores indicating greater severity of PGP^13^. Cronbach's alpha was 0.97 in the original study and 0.91 in our study. The SF-36, whose Turkish validity and reliability were established by Koçyiğit et al.^14^, contains 36 items across eight subscales assessing various domains of quality of life. Each subscale is scored from 0 to 100, with higher scores indicating better health-related quality of life. Cronbach's alpha values for the Turkish version ranged between 0.785 and 0.974. The BDI, adapted to Turkish by Hisli^15^ comprises 21 items measuring depressive symptoms, scored from 0 to 3. Total scores range from 0 to 63, with higher scores indicating greater depressive symptom severity. The Cronbach's alpha reliability coefficient was 0.80 in the original study and 0.91 in our study. Data were collected through face-to-face interviews at times convenient for the participants. Completing all forms took approximately 15–20 min.

Data were analyzed using IBM SPSS Statistics version 26. Descriptive statistics, including frequency, percentage, mean, and standard deviation, were calculated. Normality was assessed using the Kolmogorov-Smirnov test, and Pearson's correlation was applied accordingly. Relationships between variables were examined using Pearson's correlation analysis and simple linear regression. A p<0.05 was considered statistically significant.

## RESULTS

Of the 450 individuals initially recruited, 10 (2.2%) were excluded due to incomplete data, and 7 (1.6%) did not meet the inclusion criteria, resulting in a final sample of 433 participants.

Table 1 summarizes the sociodemographic and obstetric characteristics of the pregnant women, indicating a predominance of university and above education, housewife status, nuclear family structure, and planned natural pregnancies.

**Table 1:** Distribution of pregnant women according to their descriptive characteristics (n=433).

Features		Mean (SD)	
Age		27.81 (5.12)	
Size		163.16 (5.36)	
Pre-pregnancy weight		65.52 (12.91)	
Current weight		74.19 (13.46)	
Pregnancy week		28.62 (9.21)	
Number of pregnancies		1.83 (1.05)	
Number of births		0.79 (0.94)	
Number of living children		0.75 (0.90)	
Number of miscarriages/abortions		0.27 (0.61)	
VAS (0–10 puan)		4.88 (2.24)	
Education status		**n**	**%**
	Primary education	93	21.5
	High school	169	39.0
	University and above	171	39.5
Job			
	Housewife	332	76.7
	Officer	57	13.1
	Private sector	35	8.1
	Other	9	2.1
Family type			
	Nuclear family	368	85.0
	Extended family	65	15.0
Pregnancy status			
	Single pregnancy	428	98.8
	Twin pregnancy	5	1.2
Income status			
	My income is less than my expenses	16	3.7
	My income is equal to my expenses	257	59.3
	My income is more than my expenses	160	37.0
Onset of pregnancy			
	Naturally	397	91.7
	With medical help	36	8.3
The state of wanting pregnancy			
	We wanted and planned pregnancy	329	76.0
	We were not planning it	104	24.0
Getting support during pregnancy			
	No support	53	12.2
	Partner	277	64.0
	Family members	75	17.3
	My wife's family	22	5.1
	Other	6	1.4
Smoking			
	Using	67	15.5
	Not using	366	84.5
Alcohol use			
	Using	12	2.8
	Not using	421	97.2
Gynecological surgery status			
	Yes	50	11.5
	No	383	88.5

n: number of participants; %: percentage value. VAS: Visual Analog Scale.

Table 2 indicates statistically significant positive associations among physical functioning, PRD and ERD, social functioning, pain, and general health perception. Pain also shows a positive association with general health perception, while negative associations were observed with pelvic girdle-related measures, depressive symptoms, pain severity, and gestational week averages (p<0.05). PGQ and its subscale scores were found to be positively associated with depressive symptoms, Visual Analog Scale (VAS) scores, and gestational week averages (p<0.05).

**Table 2:** Mean, standard deviation, and correlation values of Pelvic Girdle Questionnaire, Beck Depression Inventory, SF-36 Quality of Life Scale sub-dimensions, and some sociodemographic variables (n=433).

Variables	Mean (SD)	1	2	3	4	5	6	7	8	9	10	11	12	13	14
1. PF	19.52 (5.47)	–													
2. PRD	5.07 (1.43)	0.412**	–												
3. ERD	3.91 (1.15)	0.389**	0.672**	–											
4. EV	15.09 (3.68)	-0.029	-0.116*	-0.124**	–										
5. MH	18.09 (4.85)	-0.060	-0.144**	-0.132**	0.645**	–									
6. SF	6.59 (1.85)	0.213**	0.321**	0.316**	-0.234**	-0.149**	–								
7. Pain	7.03 (1.98)	0.221**	0.333**	0.234**	-0.218**	-0.175**	0.515**	–							
8. GHP	15.87 (4.31)	0.130**	0.165**	0.203**	-0.279**	-0.200**	0.310**	0.268**	–						
9. PGQ	45.75 (20.28)	-0.218**	-0.249**	-0.131**	0.147**	0.048	-0.265**	-0.502**	-0.177**	–					
10. Activity	45.25 (21.57)	-0.172**	-0.206**	-0.090	0.125**	0.017	-0.218**	-0.430**	-0.139**	0.981**	–				
11. Symptom	47.74 (23.68)	-0.309**	-0.318**	-0.246**	0.173**	0.141**	-0.341**	-0.585**	-0.250**	0.708**	0.558**	–			
12. BDI	15.91 (11.39)	-0.030	-0.060	-0.040	0.304**	0.196**	-0.401**	-0.347**	-0.361**	0.217**	0.176**	0.289**	–		
13. VAS	4.88 (2.24)	-0.154**	-0.162**	-0.127**	0.081	0.032	-0.219**	-0.481**	-0.236**	0.451**	0.376**	0.562**	0.227**	–	
14. Pregnancy week	28.62 (9.21)	-0.246**	-0.131**	-0.191**	-0.030	0.004	0.035	-0.117*	0.065	0.136**	0.096*	0.232**	-0.040	0.121*	–

Pearson's correlation coefficient was used. SD: standard deviation; PF: physical function; PRD: physical role difficulty; ERD: emotional role difficulty; EV: energy/vitality; MH: mental health; SF: social functioning; GHP: general health perception; PGQ: Pelvic Girdle Questionnaire; BDI: Beck Depression Inventory; VAS: Visual Analog Scale.

* p<0.05,

** p<0.01.

According to Table 3, in the model established for pregnant women, the physical function (PF) had a significant effect on PGQ (p=0.045). Physical role difficulty (PRD) had a significant effect on PGQ (p=0.035), and a one-unit increase in PRD was associated with a decrease of 1.709 in PGQ. Furthermore, the Visual Analog Scale (VAS) had a statistically significant effect on PGQ (p<0.001). For each one-unit increase in PGQ, VAS increased by 2.368. The PGQ in pregnant women is explained by PF, PRD, emotional role difficulty (ERD), energy/vitality (EV), mental health (MH), pain, number of births, and VAS, accounting for 32.9% of the total variance in PGQ scores. These findings indicate that factors influencing the severity of PGP in pregnant women are related to both physiological and psychological dimensions.

**Table 3:** Factors affecting Pelvic Girdle Questionnaire scores according to linear regression analysis: the role of SF-36 quality of life subscales, Beck Depression Inventory, and sociodemographic variables.

	β^1^ (95%CI)	β^2^	t	p
Constant	101.115 (49.749/152.482)		3.870	**<0.001**
PF	-0.336 (-0.665/-0.007)	-0.091	-2.009	**0.045**
PRD	-1.709 (-3.293/-0.125)	-0.121	-2.121	**0.035**
ERD	1.963 (0.025/3.901)	0.111	1.991	**0.047**
EV	0.755 (0.161/1.349)	0.137	2.497	**0.013**
MH	-0.482 (-0.914/-0.050)	-0.115	-2.192	**0.029**
SF	0.250 (-0.850/1.350)	0.023	0.447	0.655
Pain	-4.368 (-3.226/-2.228)	-0.323	-6.059	**<0.001**
GHP	-0.013 (-0.407/0.433)	0.003	0.063	0.950
Age	-0.231 (-0.573/0.112)	-0.058	-1.325	0.186
Pre-pregnancy weight	0.175 (-0.425/0.074)	-0.112	-1.380	0.168
Size	-0.186 (-0.502/0.130)	-0.049	-1.158	0.247
Current pregnancy weight	-0.064 (-0.176/0.305)	-0.043	0.524	0.601
Number of pregnancies	1.749 (-1.999/5.497)	0.091	0.917	0.360
Number of births	-6.454 (-12.401/-0.507)	-0.300	-2.133	**0.033**
Number of living children	5.243 (-1.124/11.610)	0.232	1.619	0.106
Number of miscarriages/abortions	-1.919 (-5.704/1.867)	-0.058	-0.996	0.320
VAS	2.368 (1.529/3.207)	0.261	5.546	**<0.001**
BDI	0.079 (-0.089/0.246)	0.044	0.922	0.357

F=12.748; p<0.001; R²=0.329; Standard error of estimate=16.616; β^1^: unstandardized coefficient; β^2^: standardized coefficient; BDI: Beck Depression Inventory; CI: confidence interval; PF: physical function; PRD: physical role difficulty; ERD: emotional role difficulty; EV: energy/vitality; MH: mental health; SF: social functioning; GHP: general health perception; VAS: Visual Analog Scale; Durbin-Watson=1.753. Bold values in the column indicate statistical significance (p<0.05).

## DISCUSSION

This study examined the impact of PGP on quality of life and depressive symptoms in pregnant women. This study found a positive correlation between PF and role limitations, social function, pain, and health perception, and a negative correlation with PGQ. This result is also emphasized in the study by Karabulutlu and Çiçek^16^. PGP limits pregnant women's physical functioning, making daily activities more challenging^17^. This study found that as pregnancy progresses, pain increases and PF decreases, with PGP sufferers having worse postural balance, especially in the third trimester^18^. This condition is thought to have a profound impact on the psychological well-being of pregnant women, in addition to their physical challenges, and early intervention is considered crucial.

This study found a positive correlation between role difficulty, social function, pain, and health perception, and a negative correlation with energy, MH, PGQ, VAS scores, and gestational age. These results are consistent with those of Karabulutlu and Çiçek^16^. Similarly, Gashaw et al.^19^ found that more than half of pregnant women with PGP had moderate to severe activity limitations that affected their physical functioning and health. The study found a positive relationship between ERD, social function, and pain, and a negative relationship with energy, MH, PGQ, VAS, and gestational age. Similar findings were reported by Karabulutlu and Çiçek in their study examining the quality of life of pregnant women. Algård et al.^20^ found that early depressive symptoms increase the likelihood of severe PGP later in pregnancy. Psychosocial interventions providing emotional support should be integrated with PGP management.

In our study, social function and pain were positively correlated with health perception and negatively correlated with PGQ, depression, and VAS. A meta-analysis by Halliday et al.^21^ supported our findings, linking depression to PGP, and nurses and midwives can improve health by relieving PGP through support and management. This condition significantly affects pregnant women's psychological well-being, underscoring the need for early intervention.

Our study found a negative relationship between pain and PGQ, depression, VAS, and gestational age. These findings are consistent with Robinson et al.^17^ findings on quality of life in pregnant women and Algård et al.^20^ findings on depression during pregnancy. The strong correlation between PGQ, depression, and pain highlights PGP's significant impact on physical and psychological well-being during pregnancy. Eroğlu and Karataş^22^ found that PGP increased pain, lowered health levels, and negatively impacted quality of life due to anxiety and depression. Health perception critically affects pain, depression, and quality of life in pregnancy.

The effect of PRD on PGQ in pregnant women was found, supported by previous research^17^. Specifically, each 1-point increase in PGQ score resulted in a 1.709-point decrease in PRD. Additionally, ERD influences PGQ, suggesting that emotional well-being in pregnant women may be compromised, with energy levels also impacting PGQ scores^17^.

The effect of MH on PGQ in pregnant women suggests that PGP negatively affects MH, with pain significantly affecting PGQ^17^. A significant effect of the number of births on PGQ scores was identified, suggesting that previous births may contribute to PGP development, as reported by Bjelland et al^23^.

The total PGQ significantly influenced the VAS, with each one-unit increase in PGQ resulting in a 2.368-point increase in VAS. Moreover, 32.9% of the variance in PGQ scores was accounted for by the PF, PRD, ERD, EV, MH, and pain subscales, along with the number of births and VAS. Pain in pregnant women is linked to physical condition, emotional well-being, energy, and previous childbirth experiences.

## LIMITATIONS OF THE STUDY

This study's findings are inherently limited in generalizability due to its single-center, cross-sectional design. Pain levels were assessed via self-report, which may introduce recall bias. Furthermore, the exclusion of critical psychosocial variables such as anxiety, stress, coping mechanisms, and social support restricts the comprehensiveness of the results. Future multicenter, longitudinal studies incorporating these essential factors are warranted to substantially enhance the validity and clinical applicability of the findings.

## CONCLUSION

Pelvic girdle pain during pregnancy has been found to significantly impair physical functioning, emotional well-being, energy levels, and MH, thereby increasing pain and contributing to mobility limitations, which collectively diminish overall quality of life. Furthermore, PGP severity increases with gestational age and is associated with higher pain perception and depressive symptoms. These findings highlight the important role of nurses and midwives in monitoring, supporting, and educating pregnant women throughout this process to help manage symptoms and maintain well-being.

## Data Availability

The datasets generated and/or analyzed during the current study are available from the corresponding author upon reasonable request.
